# Mobile Reading Attention of College Students in Different Reading Environments: An Eye-Tracking Study

**DOI:** 10.3390/bs15070953

**Published:** 2025-07-14

**Authors:** Siwei Xu, Mingyu Xu, Qiyao Kang, Xiaoqun Yuan

**Affiliations:** 1 School of Information Management, Wuhan University, Wuhan 430072, China; 2022301042027@whu.edu.cn (S.X.); 2022301042065@whu.edu.cn (M.X.); 2 Intelligent Computing Laboratory for Cultural Heritage at Wuhan University, Wuhan 430072, China; kang_qiyao@163.com

**Keywords:** reading environment, mobile reading, attention, eye-tracking experiments

## Abstract

With the widespread adoption of mobile reading across diverse scenarios, understanding environmental impacts on attention has become crucial for reading performance optimization. Building upon this premise, the study examined the impacts of different reading environments on attention during mobile reading, utilizing a mixed-methods approach that combined eye-tracking experiments with semi-structured interviews. Thirty-two college students participated in the study. Quantitative attention metrics, including total fixation duration and fixation count, were collected through eye-tracking, while qualitative data regarding perceived environmental influences were obtained through interviews. The results indicated that the impact of different environments on mobile reading attention varies significantly, as this variation is primarily attributable to environmental complexity and individual interest. Environments characterized by multisensory inputs or dynamic disturbances, such as fluctuating noise and visual motion, were found to induce greater attentional dispersion compared to monotonous, low-variation environments. Notably, more complex potential task-like disturbances (e.g., answering calls, conversations) were found to cause the greatest distraction. Moreover, stimuli aligned with an individual’s interests were more likely to divert attention compared to those that did not. These findings contribute methodological insights for optimizing mobile reading experiences across diverse environmental contexts.

## 1. Introduction

With the rapid development of digital intelligence technologies, an increasing number of individuals are opting to use electronic devices such as computers and smartphones for reading (Liao et al., 2024). Among these devices, mobile platforms—particularly smartphones—have become the predominant reading medium due to their portability and constant availability, enabling reading to naturally integrate into fragmented moments throughout daily routines. Consequently, mobile reading has evolved into an integral aspect of contemporary life, occurring seamlessly across diverse contexts. This widespread adoption is underscored by statistics: in China, for instance, the proportion of adults engaging in digital reading reached 80.3%, and mobile reading penetration stood at 78.3% in 2023 (Xinhua, 2024); similarly, in the United States, 86% of adults reported reading on smartphones, computers, or tablets “often” or “sometimes” (Shearer, 2021). Among various demographic groups, young adults, particularly college students, demonstrate a greater propensity for mobile reading, driven by their openness to new technologies, availability of time, and higher energy levels compared to older or less-educated cohorts (L. Zhang & Ma, 2011; Xie, 2018). Therefore, investigating mobile reading of college students may hold substantial value for both academic research and practical application.

Mobile reading, enabled by anytime-anywhere access through diverse terminal devices, inherently generates a wide range of physical reading environments. These physical environments—defined as the actual locations and their ambient conditions in which reading occurs (e.g., classrooms or libraries) (Kuzmičová, 2016; Vogrinčič Čepič, 2019)—do not merely serve as passive settings but actively shape the reading experience. Reading itself is a cognitively demanding task that requires sustained attention, efficient information processing, and continuous comprehension. However, the diversity of mobile reading environments—ranging from low-distraction settings to contexts with complex combinations of potential disturbances (e.g., background noise, physical movement, visual distractions)—may differentially impact these cognitive processes and reading performance (Lin & Huang, 2013; Thompson et al., 2012; H. Zhang et al., 2018; Meschtscherjakov et al., 2019). While conducive environments enhance attentional engagement and facilitate better outcomes, adverse conditions may disrupt focus and hinder comprehension (Kuzmičová, 2016). To mitigate such environmental effects, readers often adopt strategies to sustain attention and optimize reading performance. Therefore, systematically investigating how environments affect attention during mobile reading is essential for deepening our understanding of mobile reading behaviors and for guiding the development of evidence-based strategies that support effective reading across varied real-world contexts.

Previous research has extensively explored factors such as text size, text spacing, and interface design in relation to mobile reading effectiveness, yielding valuable findings (Antona et al., 2018; J. Li et al., 2021; G. Hou et al., 2020; Y. Li et al., 2023). However, relatively limited attention has been paid to the role of environmental context in shaping mobile reading experiences. Therefore, this study aims to address this gap by systematically examining the impacts of diverse reading environments on attention during mobile reading, thereby advancing theoretical understanding of mobile reading behaviors and providing evidence-based practical guidance. To this end, seven different external disturbances (including noise, verbal communication, and potential task-like disturbances) and eight different external scenes (such as a library, a classroom, and public bus) commonly encountered in life were selected as reading environments in the experiment to investigate their impacts on attention and reading performance. Following this, a combination of eye-tracking experiments and post-experiment interviews was adopted to comprehensively analyze these effects. Based on the results, a set of practical strategies was proposed to assist mobile readers in maintaining attention and achieving favorable reading performance across varying environmental contexts.

## 2. Literature Review

### 2.1. Factors Impacting Mobile Reading Performance

Mobile reading is defined as the practice of accessing, receiving, or downloading electronic content via portable devices—including smartphones, tablets, and e-readers—through wireless or mobile communication networks (Ye & Liu, 2021). While retaining its fundamental nature as a cognitive activity for information acquisition or knowledge construction, mobile reading diverges from print-based reading across multiple dimensions—such as technological mediation, interactivity patterns, and environmental dynamism—which collectively reshape cognitive and behavioral engagement (Shimray et al., 2015; J. Hou et al., 2017). Against this backdrop, understanding factors that influence mobile reading performance—particularly those affecting cognitive processing efficiency and comprehension outcomes—has become a central focus in recent research.

Among these, text-related variables have attracted the most scholarly attention (Nurmahanani, 2024; Y. Li et al., 2023; He et al., 2019; G. Hou et al., 2020; J. Li et al., 2021). For instance, Nurmahanani (2024) demonstrated that the presentation form of texts significantly affected students’ reading comprehension, with mixed display imposing the highest cognitive load. Y. Li et al. (2023) found that entertainment texts were associated with better comprehension than scientific texts across varied environments. He et al. (2019) and G. Hou et al. (2020) utilized eye-tracking methods to investigate layout and typography, revealing that variations in font size, spacing, and line height significantly impact reading efficiency and experience, particularly for elderly readers. J. Li et al. (2021) further extended this line of inquiry by comparing interaction modes, showing that paging mode induces less visual fatigue than scrolling for both long and short texts.

In addition to text-related factors, some studies have examined the role of physical posture in shaping reading outcomes. Nurmahanani (2024) observed that sitting enhanced sustained attention more effectively than standing or walking, but comprehension of memory-based items was better while walking than sitting. Yang et al. (2020) reported higher learning achievement in sitting and standing postures compared to moving postures. Similarly, Kong et al. (2025) quantified the impact of walking specifically, finding that it significantly degraded reading performance on mobile devices compared to sitting.

Although these studies highlight critical factors influencing mobile reading, investigations into the role of the surrounding physical environment remain limited. A few exceptions exist: Antona et al. (2018) found greater visual fatigue when reading on smartphones in dark environments compared to bright ones, while Y. Li et al. (2023) used an Electroencephalogram (EEG) to demonstrate better reading performance in quiet settings compared to distracting ones. However, these findings typically address environmental conditions in isolation—such as noise or lighting—without considering the complex and dynamic nature of real-world mobile reading environments. Obviously, while prior research has made substantial contributions in identifying how text format, posture, and individual environmental elements affect mobile reading, there remains a critical gap in understanding how the environment, as an integrated and variable system, influences attention and comprehension during mobile reading. Addressing this gap is essential for developing valid insights and designing interventions that enhance reading performance in diverse mobile contexts.

### 2.2. Attention as a Core Mechanism in Mobile Reading

Attention has been widely examined in digital reading research as a key factor influencing comprehension and learning outcomes. Delgado and Salmerón (2021) found that screen reading led to reduced attention and, to some extent, shallow information processing and lower comprehension. Further specifically in the mobile context, Jeong (2012), using multiple assessment methods including critical flicker-fusion frequency (CFF) measurements, comprehension tests, and questionnaires, reported that students performed better when reading paper books than e-books, attributing the difference to the higher attentional demands and error proneness of screen reading. Y. Li et al. (2023) showed that reading in a quiet environment improved both memory recall and comprehension, highlighting the role of external conditions in sustaining attention. C. Chen and Lin (2014) and Nurmahanani (2024) found a significant positive correlation between sustained attention and reading comprehension in the high reading comprehension group. These findings collectively underscore that attention is not only a measurable cognitive construct but also a robust predictor of reading performance.

The theoretical basis for the role of attention in reading lies in its close relationship with memory systems. Attention reflects the process in which human cognitive activities continuously focus on selective objects or respond discretely to specific stimuli, that is, sustained attention and focused attention (Cao et al., 2024). Moreover, the memory system comprises two core components: working memory, which temporarily stores and processes fragments of information from the text to form a coherent understanding, and long-term memory, which stores processed information for an extended period (Cowan, 2014). New information must be processed in working memory before it can be stored in long-term memory (Paas & Van Merriënboer, 2020). Critically, increasing evidence shows that working memory depends on the moment-to-moment state of attention: heightened attentional engagement enhances working memory efficiency (Adam & deBettencourt, 2019), which in turn supports more effective integration and retention of textual information. Given this connectedness, attention serves as a valid and sensitive framework for assessing how different reading environments impact cognitive performance. As such, it offers a theoretically grounded and empirically supported lens through which to understand variations in mobile reading outcomes.

### 2.3. Assessing Attention During Reading with Eye-Tracking

Eye-tracking is an experimental method for recording eye movement and gaze position over time and across different tasks, primarily utilizing specialized eye trackers. Most modern eye trackers typically operate by capturing light reflections from the cornea and retina. Under conditions of relative stability in both the light source and head position, these devices analyze the spatial relationship between these reflections to derive precise data of pupil orientation (Carter & Luke, 2020).

A growing body of research has highlighted the strong physiological and functional connection between eye movements and the nervous system (Bell, 1823; Tanabe et al., 2002), positioning eye movements as both reflections of neural activity and valuable windows into underlying cognitive processes. Thus, eye-tracking technology has emerged as a robust and objective method for examining brain function, particularly in the context of cognitive, attentional, and information processing mechanisms. Armstrong and Olatunji (2012) suggested that eye-tracking technology provided a relatively direct and continuous measure of explicit visual attention and was an important complement to reaction time measures in the study of attentional biases in patients with affective disorders. Similarly, Rahal and Fiedler (2019) argued that compared to traditional methods, eye-tracking is a cost-effective and accurate tool for studying cognitive and emotional processes behind human behavior in social settings. The versatility of eye-tracking has also been evidenced by its diverse applications across research domains. In clinical psychology, it has been used to study attentional biases in affective disorders (Armstrong & Olatunji, 2012) and attentional control deficits in phobic individuals (Saalwirth et al., 2024). Human-computer interaction research employs it to assess attention allocation in driving simulations (Merat et al., 2014) and online advertising (Peker et al., 2021). Educational studies leverage it to evaluate learning under varied conditions (Rappa et al., 2019) and infographic comprehension (Wu & Liu, 2024). Additionally, it serves as a fundamental tool in basic cognitive research on reading processes (Jordan et al., 2016), scene perception (Stapel et al., 2020), visual search (Hsiao et al., 2021), paradoxical intuition (Rosner et al., 2022), domain-specific expertise (Reingold & Sheridan, 2023), and social media engagement (Kohout et al., 2023), demonstrating its broad utility.

Specifically, eye-tracking research primarily relies on metrics derived from fixations and saccades. A fixation occurs when the eye remains relatively stationary for a brief period to process visual information, while saccades are defined as the rapid ballistic movements between fixations during which visual input is suppressed. Consequently, fixation-based metrics provide the primary window into cognitive processing dynamics, while saccadic metrics primarily reflect visual exploration strategies (Rayner, 2001; Lai et al., 2013). Key fixation-related metrics include total fixation duration (the cumulative time spent fixating on specific Areas of Interest, AOIs), average fixation duration (the mean duration of fixations on each AOI), fixation count (the total number of fixations on a specific AOI), and time to first fixation (the time elapsed from stimulus onset to the first fixation on a target AOI). Crucially, longer fixation durations and increased fixation frequency are robustly associated with higher cognitive workload, more complex information processing, and deeper cognitive effort, whereas time to first fixation serves as a sensitive marker of visual search efficiency and the initial attentional capture by specific aspects of the scene. Saccadic metrics primarily include saccade count (the total number of saccades counted within an AOI), saccadic amplitude (the distance covered by a saccade, reflecting the scope of information search), and saccade regressions (backward saccades to previously viewed areas; particularly significant in reading, these are often associated with comprehension difficulties or information re-processing). In summary, fixation metrics (notably total duration and count) provide key indicators of cognitive load, attentional engagement, and processing depth. Saccadic metrics, conversely, elucidate the underlying structure and efficiency of visual search and information acquisition strategies.

Therefore, by extracting data such as fixation points, fixation duration and count, and saccade distance from eye movement recordings, researchers can study individuals’ visual attention allocation and intrinsic cognitive processes. For instance, Peker et al. (2021) investigated visual attention to online banner ad elements using eye-tracking. They found images attracted the most attention, evidenced by significantly higher fixation count (FC), longer total visit duration (TVD), and shorter time to first fixation (TTF). Conversely, brands received the least attention (lowest FC/TVD). Similarly, Wu and Liu (2024) examined college students’ infographic reading using eye-tracking. High-score comprehenders showed significantly greater total fixation duration (TFD), higher TFD ratios on graphs, and more transitions between words and graphs, indicating deeper integrative processing. In contrast, low-score readers exhibited higher TFD ratios on words and larger saccade amplitudes, suggesting inefficient information-searching behavior. Dunaway et al. (2018) employed eye-tracking across devices to reveal that mobile users exhibited significantly shorter news content fixation durations and fewer link fixations (lower counts/durations) versus computer users. These metrics confirm attenuated visual attention on smaller screens, attributed to higher cognitive costs of systematic processing in mobile settings.

Obviously, eye-tracking technology provides a powerful methodological alternative to traditional self-report measures and post-hoc tests. It provides continuous, real-time, and objective data on attentional allocation, cognitive load, and information acquisition efficiency through quantitative metrics such as total fixation duration and fixation count, offering unparalleled insight into ongoing cognitive processes. This capability is particularly valuable for mobile reading research, where it enables researchers to monitor attention allocation and cognitive processes when individuals interact with text across dynamic and varied environments (Y. Chen et al., 2023). Thus, eye-tracking is exceptionally well-suited for investigating how environmental factors influence attention during mobile reading, generating precise data that can advance both theoretical understanding and practical applications.

### 2.4. Present Study

#### 2.4.1. Research Gap and Questions

The literature review mentioned above reveals a critical gap in current research: while substantial progress has been made in understanding the impact of text-related factors (e.g., format, layout, typography) and physical posture on mobile reading performance, and while attention has been established as a key cognitive mechanism linking environmental factors to comprehension through working memory, there remains a lack of investigation into how surrounding physical environments influence reading. Most existing studies on environmental impacts have focused on decontextualized single factors (such as lighting or noise) in static conditions, failing to address the complex, integrated, and diverse nature of real-world mobile reading scenarios, where multiple environmental factors converge into combinations (e.g., auditory and visual distractions interact simultaneously). Additionally, although attention is widely acknowledged as a critical predictor of reading outcomes and eye-tracking is recognized as a reliable, real-time tool for measuring attentional focus, no research has yet used eye-tracking to examine how holistic environmental contexts influence attention during mobile reading. This represents a significant gap in both theoretical knowledge and practical design recommendations.

To address this critical gap, this study primarily employs eye-tracking to investigate how different mobile reading environments—characterized by combined disturbances such as noise, motion, and visual stimuli—influence attention allocation. Specifically, it focuses on the following two research questions:

Q1: Do significant differences occur in attention distribution across various mobile reading environments? That is, do these environments exert differential impacts on attention during mobile reading?

Q2: What key factors primarily give rise to these differential impacts on attention?

#### 2.4.2. Theoretical Framework and Hypotheses

Kahneman’s (1973) resource limitation theory conceptualizes attention as a finite cognitive resource essential for processing stimuli. Due to its limited nature, individuals must selectively allocate their attention to specific information while suppressing irrelevant stimuli. This selective allocation occurs through two synergistic attention functions: (1) bottom-up (exogenous) attention, which is an automatic, externally driven process triggered by salient stimuli like sudden noises or high-contrast visuals, and (2) top-down (endogenous) attention, which is a voluntary, internally controlled process that enables individuals to prioritize task-relevant information (Corbetta & Shulman, 2002; Katsuki & Constantinidis, 2014). Both the salience of stimuli and the individual’s volitional control are crucial in determining how attention is allocated. On the other hand, Kahneman (1973) also posits that more complex stimuli or cognitively demanding tasks require more attentional resources. Meanwhile, the cognitive system employs a flexible resource allocation mechanism, directing more cognitive resources toward stimuli perceived as more important or engaging. Taken together, these theoretical frameworks suggest that the impact of external disturbances on attention depends on the complexity and personal relevance of the stimuli. Disturbances that are more complex or engaging are more likely to divide attention, leading to reduced reading performance.

Building on the discussion of eye-tracking introduced in Section 2.3, this study employs two well-established eye-tracking metrics—total fixation duration (TFD) and fixation count (FC)—to empirically assess attention allocation during mobile reading. Previous research (Rayner, 2001) and more recent studies (Peker et al., 2021; Wu & Liu, 2024; Dunaway et al., 2018) have demonstrated that TFD and FC are reliable indicators of sustained attentional engagement and information processing depth. These metrics align with the current study’s focus on how environmental factors disrupt cognitive engagement. Metrics related to visual search (e.g., saccade amplitude) or initial orienting (e.g., time to first fixation) were excluded from analysis due to their limited relevance to sustained engagement. Notably, while previous studies have applied TFD and FC to measure attention influenced by factors such as stimulus features (e.g., advertisement visuals, text-graphic layouts) or device context, our study aims to examine how attention is modulated by external environmental factors. Specifically, a reduction in fixation duration and count—indicating diminished attentional engagement—will be interpreted as a consequence of greater environmental interference.

**Figure 1:**
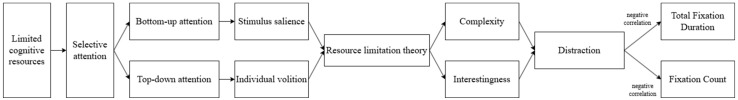
Theoretical Framework.

Incorporating Kahneman’s theory of resource limitation, the functions of selective attention, and the chosen eye-tracking metrics, our research framework is depicted in Figure 1. Based on this framework, we propose the following hypotheses:

**H1.**  *Attention allocation differs significantly across mobile reading environments, indicating that these environments exert differential impacts on attentional engagement during mobile reading.*

**H2.**  *The differences in attention allocation are primarily driven by the complexity of environmental stimuli and the reader’s interest. Specifically, more complex environmental disturbances are more likely to split attention than simpler ones, and stimuli that interest the reader are more likely to divert attention than those that do not.*

## 3. Materials and Methods

### 3.1. Method Framework

**Figure 2:**
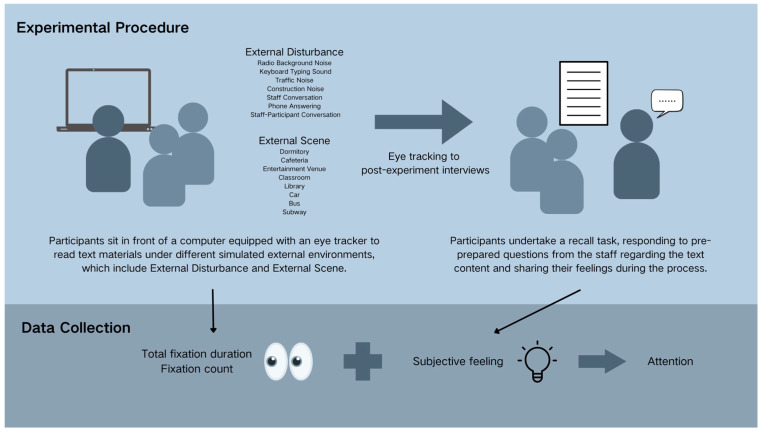
Method Framework.

The method framework was shown in Figure 2, which consisted of two major components: Experimental Procedure and Data Collection. The experimental procedure consisted of an eye-tracking experiment and a post-experiment interview. Firstly, the eye-tracking experiment required participants to sit in front of a computer equipped with an eye tracker to read text materials under different simulated external environments, which included external disturbances and external scenes. During this process, we collected the eye movement data from the participants in each external environment, specifically including total fixation duration and fixation count. Secondly, the post-experiment interviews involved participants undertaking a recall task, wherein they responded to pre-prepared questions from the staff concerning the text content and shared their feelings during the reading process. In this phase, we gathered participants’ subjective feelings. By integrating the objective eye-tracking data with the subjective feedback, we were able to analyze and discuss the specifics of readers’ attention under different mobile reading environments.

### 3.2. Participants

A total of 32 college students (17 females and 15 males; mean age = 21 years) voluntarily participated in this study. Complete demographic characteristics are presented in Table 1, including detailed distributions of gender, age, and grade. All participants had normal or corrected-to-normal vision and reported no prior experience with similar experimental tasks. Participant variability was minimal, and individuals were instructed to maintain their habitual reading practices throughout the experiment. Differences in academic backgrounds and knowledge structures were acknowledged as uncontrolled confounding variables but were not systematically manipulated within the experimental design. All participants provided informed consent and received compensation upon completion of the study.

### 3.3. Materials

The experimental materials included reading materials and background materials. To ensure the validity and scientific nature of the experimental results, three sets of different reading and background materials were prepared in the preliminary stage, and 10 college students were invited to conduct a pre-test. Informed by the results, the formal experimental materials were finalized.

#### 3.3.1. Reading Materials

Four continuous long texts were selected from online sources^1^, each consisting of approximately 3000 to 5000 words and conforming to common reading habits. The texts were fictional narratives (novels) aligned with the reading difficulty level typically encountered by senior undergraduate students in humanities and social science disciplines. As our experiment was conducted continuously with many distractions, we chose long texts instead of fragmented texts, hoping that readers would gain a deeper understanding and develop their cognition, thereby enabling a more accurate assessment of their attention in the presence of different distractions.

#### 3.3.2. Background Materials

According to the purpose of the experiment, we designed external disturbances and external scenes to simulate real-world scenarios. The external disturbance experiments included five types of noise disturbance, namely Radio Background Noise, Keyboard Typing Sound, Traffic Noise, Construction Noise, and Staff Conversation, along with two types of potential task-like disturbance, namely Phone Answering and Staff-Participant Conversation (Table 2). The first four types of disturbance were reproduced by playing pre-recorded real-world environmental sounds, and the last three types of disturbance were reproduced on-site by staff. The external scenes experiment selected representative fixed locations and commuting scenes, simulating eight common mobile reading environments—Dormitory, Library, Cafeteria, Entertainment Venue, Classroom, Car, Bus, and Subway (Table 3). All external scenes were constructed by playing video recordings of authentic environments, combined with physical props and staff participation. Additionally, each sound material of the first four external disturbances lasted 1 minute, and each video material of the eight external scenes lasted 2 minutes in our experiment. Both audio and video materials were presented via a surround LED screen to enhance immersion.

### 3.4. Apparatus for Eye-Tracking

The main equipment used in our experiment included a Tobii Pro X3-120 eye tracker (Tobii, Danderyd, Sweden; sampling rate: 120 Hz), an external data processing module, namely Tobii Studio (v.3.4.7), a Dell computer, a camera, and an LED screen.

### 3.5. Procedure

This study employed a within-subjects experimental design, where each participant was exposed to all seven types of external disturbances and eight types of external scenes. The experiment was conducted individually in a specialized laboratory equipped with soundproofing to guarantee a quiet and comfortable environment for participants. Initially, participants’ positions and eye levels were adjusted to maintain 60 cm between their eyes and the computer screen to ensure accurate recording of eye movement trajectories. Following this, participants completed a standardized nine-point calibration protocol provided by Tobii Studio software. This process involved participants fixating on sequentially presented calibration points at varying positions on the screen (top-left, center, bottom-right, etc.), followed by validation trials to ensure accuracy (error tolerance ≤ 0.5°).

During the formal experiment, participants read four text materials (displayed centrally on the screen) according to a fixed sequence of simulated external environment conditions. The reading time was controlled using pre-edited audio/video materials and by the staff. Upon completion of the reading task, participants undertook a recall task, answering pre-designed questions related to the text content as well as sharing their feelings during the process. A typical interview question was: “Which scene or disturbance had the greatest impact on your attention, and what specific factors contributed to this impact?” Generally, the entire reading and interview of each participant lasted approximately 40 min and the specific experimental process is shown in Table 4.

### 3.6. Data Analysis

During the external disturbance experiment, two participants had missing data in multiple environments due to operational errors. After excluding these two participants, the analysis was based on data from 30 participants. Moreover, in the external scene experiment, four participants had missing data, which were also excluded, resulting in the analysis being based on data from 28 participants. Additionally, the Subway scene was incomplete because the staff terminated the experiment early, so the data for this scene was removed from the analysis.

Prior to formal statistical analysis, boxplots were generated for preliminary visual assessment of data distribution characteristics across experimental conditions. The subsequent data analysis process was carried out in a step-by-step manner to examine differences in eye movement metrics under various external disturbances and scenes. First, descriptive statistics for total fixation duration and fixation count were computed using SPSS 25.0, and Shapiro-Wilk normality tests were conducted to assess the distribution of the data. When the assumption of normality was met, a one-way repeated measures ANOVA was performed to determine whether significant differences existed in the eye movement indicators across different external disturbances or scenes. If the normality assumption was violated, a non-parametric Friedman test was used instead. In cases where significant differences were identified, post-hoc pairwise comparisons with Bonferroni correction were conducted to identify specific disturbances or scenes that differed significantly from each other.

## 4. Results

### 4.1. External Disturbance Experiment Results

#### 4.1.1. Total Fixation Duration

**Figure 3:**
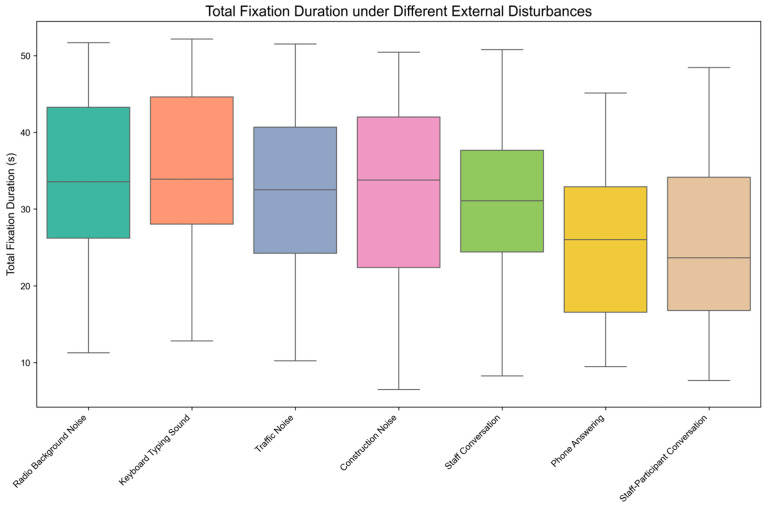
Boxplot Distribution of Total Fixation Duration under External Disturbances.

Figure 3 presents the distribution characteristics of Total Fixation Duration (TFD) across seven external disturbance conditions through boxplot visualization. Visual inspection reveals comparatively lower median TFD values under both Phone Answering and Staff-Participant Conversation conditions than under other disturbances.

The Shapiro-Wilk normality test results indicated that the total fixation duration under various external disturbances—Radio Background Noise (*p* = 0.415 > 0.05), Keyboard Typing Sound (*p* = 0.307 > 0.05), Traffic Noise (*p* = 0.535 > 0.05), Construction Noise (*p* = 0.388 > 0.05), Staff Conversation (*p* = 0.398 > 0.05), Phone Answering (*p* = 0.084 > 0.05), Staff-Participant Conversation (*p* = 0.116 > 0.05)—followed a normal distribution. Therefore, the one-way repeated measures ANOVA was considered appropriate for the analysis. Mauchly’s test of sphericity indicated that the assumption of sphericity was violated (Mauchly’s W = 0.062, *p* < 0.001). Consequently, the Greenhouse–Geisser correction was applied in the within-subjects effects analysis to adjust the degrees of freedom.

Descriptive statistics and the one-way repeated measures ANOVA results are shown in Table 5, which indicate that the impact of different external disturbances on total fixation duration is significant, F (3.063, 88.829) = 21.219, *p* < 0.001, η^2^_p_ = 0.423. This significant result suggests that different external disturbances had significantly varying levels of distraction on attention during mobile reading, as measured by TFD.

The results of post-hoc pairwise comparisons with Bonferroni correction are shown in Table 6. From Table 6, we can conclude the following consequences:

The total fixation duration under Traffic Noise was significantly shorter than that under Keyboard Typing Sound (t (174) = −3.566, *p* < 0.05, d = −0.194, 95% CI [−4.274, −0.148]). This suggests that Traffic Noise may, to some extent, cause greater attentional distraction during mobile reading than Keyboard Typing Sound, as primarily reflected by reduced TFD.

The total fixation duration under Construction Noise was significantly shorter than that under Radio Background Noise (t (174) = −3.616, *p* < 0.05, d = −0.264, 95% CI [−6.18, −0.256]) and Keyboard Typing Sound (t (174) = −3.821, *p* < 0.05, d = −0.215, 95% CI [−4.911, −0.338]). This suggests Construction Noise had a stronger disruptive effect on attentional focus than either Radio Background Noise or Keyboard Typing Sound, while this interpretation is based solely on TFD data.

Under Phone Answering disturbance, total fixation duration was significantly shorter than under Radio Background Noise (t (174) = −7.247, *p* < 0.001, d = −0.829, 95% CI [−13.547, −5.019]), Keyboard Typing Sound (t (174) = −7.346, *p* < 0.001, d = −0.776, 95% CI [−12.629, −4.751]), Traffic Noise (t (174) = −4.698, *p* < 0.05, d = −0.579, 95% CI [−11.069, −1.89]), Construction Noise (t (174) = −4.564, *p* < 0.05, d = −0.542, 95% CI [−10.49, −1.64]), and Staff Conversation (t (174) = −5.326, *p* < 0.001, d = −0.571, 95% CI [−10.385, −2.396]).

Under Staff-Participant Conversation disturbance, total fixation duration was significantly shorter than under Radio Background Noise (t (174) = −5.609, *p* < 0.001, d = −0.705, 95% CI [−13.247, −3.379]), Keyboard Typing Sound (t (174) = −5.530, *p* < 0.001, d = −0.654, 95% CI [−12.367, −3.073]), Traffic Noise (t (174) = −3.740, *p* < 0.05, d = −0.467, 95% CI [−10.412, −0.607]), Construction Noise (t (174) = −3.660, *p* < 0.05, d = −0.432, 95% CI [−9.73, −0.461]), and Staff Conversation (t (174) = −4.722, *p* < 0.05, d = −0.460, 95% CI [−9.243, −1.598]). Together, these results suggest that from TFD data, both Phone Answering and Staff-Participant Conversation caused significantly greater attentional distraction than all other disturbances, positioning them as the most disruptive conditions.

No significant differences in total fixation duration were found between the remaining disturbance groups, such as Radio Background Noise versus Keyboard Typing Sound, implying comparable levels of attentional distraction during mobile reading for these specific disturbances based on TFD measurements.

#### 4.1.2. Fixation Count

**Figure 4:**
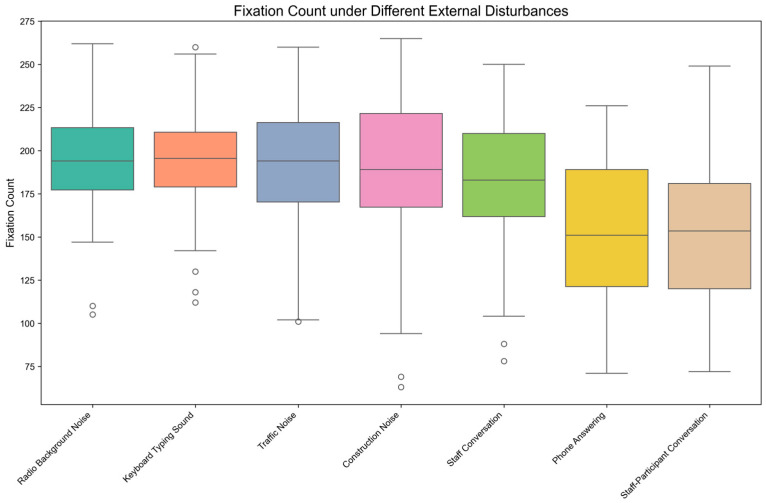
Boxplot Distribution of Fixation Count under External Disturbances.

Figure 4 presents the distribution characteristics of Fixation Count (FC) across seven external disturbance conditions through boxplot visualization. Visual inspection reveals comparatively lower median FC values under both Phone Answering and Staff-Participant Conversation conditions than under other disturbances.

The Shapiro-Wilk normality test results indicated that the fixation count under various external disturbances—Radio Background Noise (*p* = 0.289 > 0.05), Keyboard Typing Sound (*p* = 0.167 > 0.05), Traffic Noise (*p* = 0.179 > 0.05), Construction Noise (*p* = 0.063 > 0.05), Staff Conversation (*p* = 0.154 > 0.05), Phone Answering (*p* = 0.471 > 0.05), Staff-Participant Conversation (*p* = 0.784 > 0.05)—followed a normal distribution. Therefore, the one-way repeated measures ANOVA was considered appropriate for the analysis. Mauchly’s test of sphericity indicated that the assumption of sphericity was violated (Mauchly’s W = 0.089, *p* < 0.001). Consequently, the Greenhouse–Geisser correction was applied in the within-subjects effects analysis to adjust the degrees of freedom.

Descriptive statistics and the one-way repeated measures ANOVA results are shown in Table 7, which indicate that the impact of different external disturbances on fixation count is significant, F (3.341, 96.895) = 23.823, *p* < 0.001, η^2^_p_ = 0.451. This significant result also suggests that different external disturbances had significantly varying levels of distraction on attention during mobile reading, as measured by FC.

The results of post-hoc pairwise comparisons with Bonferroni correction are shown in Table 8. From Table 8, we can conclude the following consequences:

The fixation count under Staff Conversation disturbance was significantly lower than under Keyboard Typing Sound (t (174) = −4.120, *p* < 0.05, d = −0.326, 95% CI [−25.134, −2.666]). This suggests that Staff Conversation may, to some extent, cause greater attentional distraction during mobile reading than Keyboard Typing Sound, as primarily reflected by reduced FC.

The fixation count under Phone Answering disturbance was significantly lower than under Radio Background Noise (t (174) = −5.928, *p* < 0.001, d = −0.939, 95% CI [−63.298, −17.768]), Keyboard Typing Sound (t (174) = −6.496, *p* < 0.001, d = −1.014, 95% CI [−66.198, −21.335]), Traffic Noise (t (174) = −5.666, *p* < 0.001, d = −0.936, 95% CI [−64.137, −16.663]), Construction Noise (t (174) = −4.763, *p* < 0.05, d = −0.852, 95% CI [−62.468, −11.065]), and Staff Conversation (t (174) = −4.279, *p* < 0.05, d = −0.692, 95% CI [−53.104, −6.629]).

The fixation count under Staff-Participant Conversation disturbance was significantly lower than under Radio Background Noise (t (174) = −6.646, *p* < 0.001, d = −0.936, 95% CI [−59.538, −19.795]), Keyboard Typing Sound (t (174) = −6.979, *p* < 0.001, d = −1.013, 95% CI [−63.363, −22.437]), Traffic Noise (t (174) = −5.970, *p* < 0.001, d = −0.933, 95% CI [−61.578, −17.489]), Construction Noise (t (174) = −5.672, *p* < 0.001, d = −0.848, 95% CI [−56.969, −14.831]), and Staff Conversation (t (174) = −5.056, *p* < 0.001, d = −0.685, 95% CI [−48.096, −9.904]). Similarly, analysis of FC data confirmed that both Phone Answering and Staff-Participant Conversation induced significantly greater attentional distraction than all other disturbances, establishing them as the most disruptive conditions.

No significant differences in fixation count were found between the remaining disturbance groups, such as Radio Background Noise versus Keyboard Typing Sound, implying comparable levels of attentional distraction during mobile reading for these specific disturbances based on FC measurements.

#### 4.1.3. Consolidated Findings from TFD and FC Analyses

By integrating the results of total fixation duration and fixation count, we found that different external disturbances significantly vary in their impact on attention during mobile reading, providing partial support for H1. Both Phone Answering and Staff-Participant Conversation exhibited significantly lower values on both TFD and FC compared to all other conditions, indicating that these disturbances had the greatest disruptive effect on attention during mobile reading. Additionally, Traffic Noise, Construction Noise, and Staff Conversation displayed similar effects on both TFD and FC, with no significant differences between them. However, their impact was notably greater than that of Phone Answering and Staff-Participant Conversation, yet still significantly lower than the effects of Radio Background Noise and Keyboard Typing Sound. This pattern suggests that these disturbances contribute to a moderate level of attentional distraction. Finally, Radio Background Noise and Keyboard Typing Sound showed no significant difference from each other on either TFD or FC. Crucially, their values were significantly higher than those under all other disturbance conditions. This demonstrates that these two disturbances had the smallest impact (or weakest disruptive effect) on attention during mobile reading.

### 4.2. External Scene Experiment Results

#### 4.2.1. Total Fixation Duration

**Figure 5:**
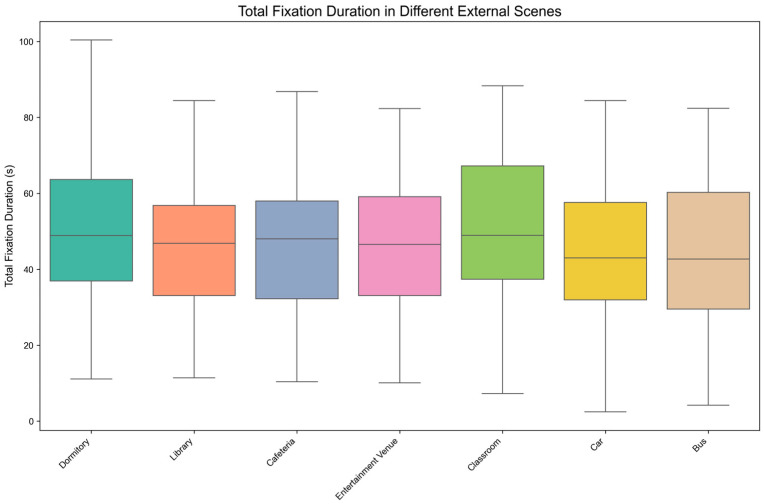
Boxplot Distribution of Total Fixation Duration under External Scenes.

Figure 5 presents the distribution characteristics of Total Fixation Duration (TFD) across seven external scene conditions through boxplot visualization. Visual inspection reveals comparatively lower median TFD values under both Car and Bus conditions than under other scenes.

The Shapiro-Wilk normality test results indicated that the total fixation duration under different external scenes—Dormitory (*p* = 0.547 > 0.05), Library (*p* = 0.513 > 0.05), Cafeteria (*p* = 0.729 > 0.05), Entertainment Venue (*p* = 0.540 > 0.05), Classroom (*p* = 0.697 > 0.05), Car (*p* = 0.949 > 0.05), Bus (*p* = 0.808 > 0.05)—followed a normal distribution. Therefore, the one-way repeated measures ANOVA was considered appropriate for the analysis. Mauchly’s test of sphericity indicated that the assumption of sphericity was violated (Mauchly’s W = 0.102, *p* < 0.001). Consequently, the Greenhouse–Geisser correction was applied in the within-subjects effects analysis to adjust the degrees of freedom.

Descriptive statistics and the one-way repeated measures ANOVA results are shown in Table 9, which indicate that the impact of different external scenes on total fixation duration is significant, F (3.039, 82.057) = 3.678, *p* = 0.015 < 0.05, η^2^_p_ = 0.120. This significant result indicates that the degree of distraction caused by different external scenes on mobile reading attention differed significantly, as measured by TFD.

The results of post-hoc pairwise comparisons with Bonferroni correction are shown in Table 10. From Table 10, we can conclude the following consequences:

The total fixation duration in the Cafeteria scene was significantly shorter than in the Dormitory scene (t (162) = −3.507, *p* < 0.05, d = −0.364, 95% CI [−13.966, −0.315]). This suggests that the Cafeteria scene induced greater attentional distraction during mobile reading compared to the Dormitory, as evidenced by reduced TFD.

The total fixation duration in the Car scene was significantly shorter than in the Dormitory scene (t (162) = −3.584, *p* < 0.05, d = −0.378, 95% CI [−14.712, −0.491]). This indicates that the Car scene also appears more disruptive to sustained attention than the Dormitory, while this interpretation is based solely on TFD data.

No significant differences in total fixation duration were found between the other scene groups, such as Cafeteria versus Entertainment Venue, implying comparable levels of attentional distraction during mobile reading for these specific scenes when measured through TFD.

#### 4.2.2. Fixation Count

**Figure 6:**
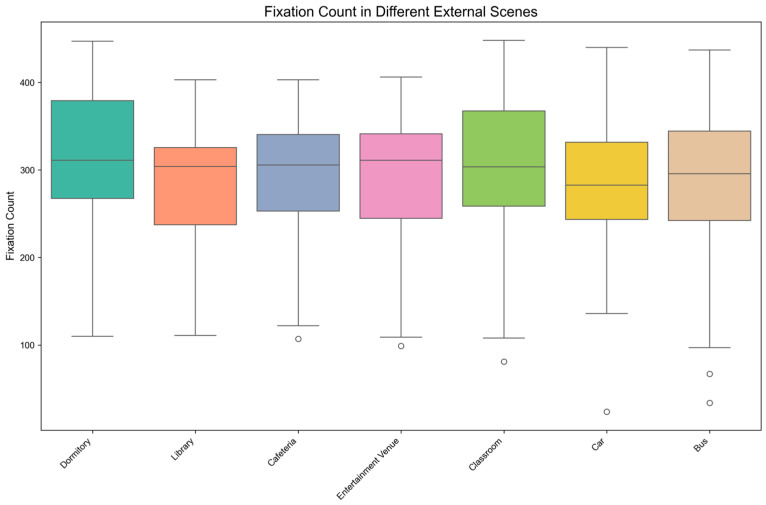
Boxplot Distribution of Fixation Count under External Scenes.

Figure 6 presents the distribution characteristics of Fixation Count (FC) across seven external scene conditions through boxplot visualization. Visual inspection indicates potential skewness in some distributions and reveals comparatively lower median FC value under the Car condition than under other scenes.

The Shapiro-Wilk normality test results indicated that fixation count followed a normal distribution in the following scenes: Dormitory (*p* = 0.314 > 0.05), Cafeteria (*p* = 0.070 > 0.05), Classroom (*p* = 0.193 > 0.05), Car (*p* = 0.335 > 0.05), Bus (*p* = 0.062 > 0.05). However, the data did not meet the assumption of normality in the Library (*p* = 0.022 < 0.05) and Entertainment Venue (*p* = 0.026 < 0.05). Therefore, a non-parametric Friedman test was conducted for further analysis.

Descriptive statistics and the Friedman test results are shown in Table 11, which indicate that the impact of different external scenes on fixation count is significant (χ^2^ (6) = 13.478, *p* = 0.036 < 0.05). This significant result indicates that the degree of distraction caused by different external scenes on mobile reading attention differed significantly, as measured by FC.

The results of post-hoc pairwise comparisons with Bonferroni correction are shown in Table 12. From Table 12, we can conclude the following consequences:

The fixation count in the Car scene was significantly lower than in the Dormitory scene (*p* < 0.05). This suggests that the Car scene induced greater attentional distraction during mobile reading compared to the Dormitory, as evidenced by reduced FC.

No significant differences in fixation count were found between the other scene groups, such as Cafeteria versus Entertainment Venue, implying comparable levels of attentional distraction during mobile reading for these specific scenes when measured through FC.

#### 4.2.3. Consolidated Findings from TFD and FC Analyses

Our combined analysis of total fixation duration and fixation count shows that external scene context exerts differing levels of attentional disruption during mobile reading, providing partial support for H1. Pairwise comparisons for both metrics revealed that only the Dormitory scene yielded a significantly higher level of attention than both the Cafeteria and Car scenes, indicating that the Dormitory environment best supports sustained, focused attention—and therefore imposes the weakest distraction. Although no other pairwise differences reached significance, the rank order of mean TFD and FC values (from lowest to highest) was Bus < Car < Cafeteria < Classroom < Entertainment Venue < Library < Dormitory. This consistent ordering across both metrics points to a graded spectrum of environmental interference, with Bus and Car settings producing the greatest distraction and the Dormitory the least.

## 5. Discussion

### 5.1. Impacts of Different External Disturbances on Attention During Mobile Reading

The results in Section 4.1 show that complex noise disturbances (Traffic Noise, Construction Noise, Staff Conversation) caused greater attentional distraction than simple noise disturbances (Keyboard Typing Sound, Radio Background Noise), which aligns with prior research findings. For instance, Mama et al. (2018) demonstrated that constant energy noise (e.g., white noise) did not impair long-term memory performance, whereas fluctuating energy noise (e.g., traffic noise) and noise containing informational fluctuations (e.g., multi-person conversations) negatively affected memory. They proposed that the fluctuations in background noise consumed additional attentional resources, thereby reducing focus on the primary task and leading to poorer performance. In our experiment, Construction Noise consisted of various complex and irregular sounds, such as tool collisions and object dragging, which are characterized by unpredictability and high amplitude variability. Similarly, Traffic Noise included continuous vehicle sounds mixed with sporadic horn blasts, featuring significant volume fluctuations and irregular patterns. Additionally, Staff Conversations exhibited both randomness and informational fluctuations, contributing to their disruptive effects. These complex and irregular auditory stimuli, due to their salient and abrupt nature, act as potent bottom-up attention grabbers (Corbetta & Shulman, 2002; Katsuki & Constantinidis, 2014), triggering involuntary shifts of attention away from the reading task. In contrast, Keyboard Typing Sound exhibited monotonous and periodic features, while Radio Background Noise had lower volume and minimal fluctuation, resulting in fewer distractions.

Notably, the mean values of total fixation duration and fixation count under Staff Conversation were lower than those under similar types of disturbances (Construction Noise and Traffic Noise). This discrepancy could be explained by the engagement of top-down attention mechanisms. Staff Conversations often contained content that triggered participants’ curiosity and interest, leading to a voluntary redirection of attentional resources away from the reading task. For instance, Participant 20 stated: “The most disruptive disturbance was staff chatting nearby, because it was interesting, I couldn’t help but listen”. In this case, the informational and engaging nature of the conversation prompted a top-down shift, where participants actively allocated their finite cognitive resources towards the personally interesting stimulus (Kahneman, 1973; Corbetta & Shulman, 2002; Katsuki & Constantinidis, 2014), further depleting resources available for reading.

Moreover, Phone Answering and Staff-Participant Conversation disturbances caused significantly greater attentional disruption than all other disturbances, establishing them as the most distracting conditions. Post-experimental interviews further substantiated these observations, with approximately 63% of participants explicitly indicating that potential task-like disturbances (Phone Answering, Staff-Participant Conversation) had a more pronounced impact on their attention than other disturbances. For instance, Participant 1 described the experience as particularly disruptive, stating: “When staff engaged me in conversation, especially when the topic was personally relevant, it forcibly pulled my attention away from the reading material”. In fact, compared to background noise disturbances, the Phone Answering and Staff-Participant Conversation disturbances were more complex. While the auditory stimuli (e.g., phone ringing, call content, or staff speech) inherently act as sensory distractors, they further impose potential task-like demands—such as answering calls or responding to conversation—which forcibly or actively divert attention allocated to the primary reading task. Concretely, since participants were unaware of the impending Phone Answering disturbance and had no opportunity to prepare for it, the sudden phone ringing immediately triggered a bottom-up attentional capture. Curiosity about the caller’s identity further compelled participants to engage with the call, representing a top-down decision to reallocate resources, leading to a marked shift in focus. As reported by Participant 7, answering the call not only disrupted attention but also deepened the distraction, with processing the content of the conversation further inhibiting the sustained focus and comprehension on the reading task. Similarly, Staff-Participant Conversation represented a more interactive and cognitively demanding form of distraction. Unlike unidirectional disturbances such as background noise, these interactions required bidirectional engagement—participants not only needed to comprehend the conversation but also formulate responses. This bidirectional engagement siphoned cognitive resources away from the reading task, impairing attention and comprehension. When the conversation topic held personal relevance, the depth of engagement increased, further amplifying the top-down resource allocation, leading participants to momentarily forget or entirely abandon the primary task.

### 5.2. Impacts of Different External Scenes on Attention During Mobile Reading

Drawing on the descriptive statistics from Section 4.2, in which mean TFD and FC values consistently ranked as Bus < Car < Cafeteria < Classroom < Entertainment Venue < Library < Dormitory, we observe a clear hierarchy of environmental distraction.

Complex, dynamic settings (Cafeteria, Classroom, Entertainment Venue) impose greater attentional disruption than simpler static scenes (Library, Dormitory). The former group shares characteristics of multisensory interference—Cafeteria scene featured fluctuating background noises (conversations, footsteps, dish clattering) and dynamic visual changes generated by moving crowds; Classroom contained auditory distractions from lectures, page-turning, writing, and visual stimuli from instructor movements; Entertainment Venue combined loud music with dynamic visual elements. The complex fluctuations and rich changes create salient features that readily trigger bottom-up attention dispersion. In contrast, the Library and Dormitory scenes maintained low-intensity auditory environments with minimal visual variations, presenting fewer salient cues to capture attention exogenously. This result is consistent with our previous conclusion that complex noise was more distracting than simple noise.

Furthermore, our findings highlight the critical role of top-down attention modulation based on personal interest, as predicted by the theoretical framework. Interviews revealed that dynamic scenes (e.g., people movement) engaged curiosity more effectively than static scenes (Participant 5), leading to voluntary shifts in attention. Previous studies also supported similar views. Ono (2018) found that in situations with many no-change stimuli, a single change stimulus attracts visual attention. Pei et al. (2022) discovered that dynamic images attract more attention than static images. Young et al. (2009) showed that when the driving task itself is relatively monotonous, novel stimuli (such as billboards) may arouse interest and attract more attention. In addition to visual aspects, background auditory stimuli also modulated attention based on personal interest. Post-experiment interviews revealed that the choice of music in the entertainment venue significantly influenced participants’ attention. Specifically, when the music aligned with personal interests, participants tended to be distracted by actively listening to it. For instance, Participant 27 stated, “The music in the Entertainment Venue was familiar to me, and because I found it interesting, I couldn’t help but get distracted, which made the interference quite significant”. This illustrates a clear case of top-down attention being voluntarily directed towards a preferred stimulus. Conversely, when the music did not arouse personal interests, it was less likely to interfere with reading. As Participant 14 noted, “The music acted more like white noise, so it didn’t distract me much”. Furthermore, in the Classroom scene, eight participants reported being easily distracted during class as they found the teacher’s lecture content more engaging than their current reading materials, again demonstrating the power of top-down allocation driven by perceived interest/relevance.

Additionally, the Car and Bus scenes caused greater distraction compared to the other five scenes. This distinction arises from dual disruption mechanisms: (1) physical shaking stimuli that required participants to divert partial attentional resources to postural control (Participant 27), thereby increasing susceptibility to attentional disruption compared to other interferences, and (2) concurrent auditory-visual disturbances (including passenger conversations, movements and externally flowing scenery) that triggered both bottom-up and top-down attention dispersion, further straining the limited pool of attentional resources (Kahneman, 1973).

### 5.3. Practical Implications

According to our findings, readers should seek out low-complexity settings—such as quiet, visually stable indoor spaces—when deep concentration is required, as these environments minimize multisensory disruptions like fluctuating noise or crowd movement. If circumstances force reading in noisy locations (for example, on public transit), employing noise-canceling headphones or earplugs may help block auditory distractions. Choosing reading materials that align with one’s interests may also strengthen top-down attentional control, making it easier to resist external interference. Finally, proactively addressing potential task-like disturbances (e.g., messages or calls) and cultivating intrinsic motivation further fortify sustained focus.

From an educational standpoint, instructional content should be matched to the learner’s environment. In high-distraction contexts (e.g., cafeterias or buses), concise “micro-modules” (e.g., <30 s) are preferable, while more complex tasks—such as critical analysis or synthesis exercises—should be reserved for stable settings like dormitories. Educational platforms can support this by offering content banks organized by duration and cognitive demand. Additionally, incorporating simple attention-training exercises (for instance, the Schulte Grid) can build learners’ foundational capacity for sustained focus, enhancing their resilience to environmental interruptions during mobile reading.

Our findings also inform mobile reading software design. Developers could integrate features like auto-saving reading progress, along with tools for highlighting and note-taking, to help users resume reading promptly after distractions. Additionally, enabling user customization of interfaces—such as adjustable font styles, background colors, progress tracking displays, and annotation tools—combined with personalized content recommendations that align with readers’ interests, may contribute to sustaining attention during mobile reading. Furthermore, adaptive design elements like night mode, outdoor mode, and anti-shake technology help users accommodate varying environmental conditions.

Collectively, our findings support the effectiveness of these strategies in reducing distractions and enhancing immersive engagement during reading tasks. Therefore, mobile readers could better maintain attention in various complex environments, ultimately improving overall reading performance.

### 5.4. Limitations and Future Directions

Despite the insights gained, several limitations warrant consideration and point toward avenues for future research. Firstly, the sample size in this study was relatively small, which not only limits the generalizability of the results, but also potentially reduces statistical power to detect smaller effects. Therefore, a larger sample size in future studies would provide more robust evidence and allow the detection of more subtle influences. Secondly, general reading performance scores were not collected prior to the experiment, limiting our ability to account for potential confounding effects of baseline reading proficiency on the measured outcomes. To deal with this, future studies could include standardized assessments of reading ability to better isolate the specific impact of external environments on mobile reading performance. Thirdly, while simulating external scenes allowed for controlled manipulation of disturbances, it sacrifices ecological validity compared to testing in actual physical environments. Thus, in the future, studies could address this by using eye-tracking either in unconstrained natural settings (maximizing ecological validity, though with reduced control) or in advanced VR/AR environments (preserving experimental control while enhancing ecological validity). Fourthly, the fixed presentation order of experimental conditions may have introduced order effects (e.g., practice or fatigue effects), potentially confounding the results. Future studies could implement randomization strategies. Lastly, our research focused on the reading performance of general texts, without examining how text difficulty level interacts with scene complexity. Therefore, future research could further explore performance differences when reading materials of different difficulties in various scenes.

## 6. Conclusions

In this study, we combined eye-tracking with post-experiment interviews to investigate the impacts of different reading environments on attention during mobile reading. Based on experimental data and discussions in Section 4 and Section 5, we confirmed hypothesis H1, demonstrating significant differences in attention levels across different external disturbances and scenes during mobile reading.

More detailed, we found that (1) in external disturbances, Phone Answering and Staff Participant Conversation had the greatest impact on mobile reading attention, followed by Traffic Noise, Construction Noise, and Staff Conversation, while Radio Background Noise and Keyboard Typing Sound had the least distraction; (2) in external scenes, Bus and Car Scenes had the greatest impact on mobile reading attention, followed by Cafeteria, Classroom, and Entertainment Venue, while Library and Dormitory had the least distraction.

The observed differences in attention allocation can be primarily attributed to two factors: environmental complexity and individual interest. Environments characterized by high volatility—such as fluctuating noise levels, dynamic visual changes, or compounded multisensory disturbances (e.g., simultaneous auditory, visual, and vibrational stimuli)—were found to induce greater attentional dispersion compared to relatively stable, low-variation settings. Particularly, disturbances that involved potential task-like demands, such as answering phone calls or engaging in conversations, were significantly more disruptive than passive background noise. These potential task-like disturbances not only triggered sensory interference but also introduced goal-directed cognitive demands, thereby exerting a more profound impact on attentional resources. In addition to environmental complexity, participants’ personal interest in the surrounding stimuli emerged as a critical moderator of attentional outcomes. When the distracting elements aligned with participants’ interests, they were more likely to voluntarily shift their attention away from the reading task, leading to active, self-initiated distraction. Conversely, in cases where participants had little to no interest in the external stimuli, they demonstrated a greater capacity to sustain attention on the reading task, effectively mitigating the influence of environmental disruptions. Taken together, these findings suggest that the extent to which various environmental conditions impact mobile reading attention is shaped by the interplay between external stimulus characteristics (e.g., auditory input, visual variation, physical motion) and readers’ intrinsic motivation and attentional control. These results provide empirical support for hypothesis H2.

Additionally, it is worth noting that whether different disturbance types can capture the reader’s interest often depends on the individual, meaning the impact of disturbances on reading is not absolute. This would also be used to explain why attention levels were not always significant in different reading disturbances or scenes in our experiments.

Overall, this study makes three pivotal contributions to advancing mobile reading research and practice. Theoretically, it provides the first eye-tracking evidence demonstrating how holistic, real-world environments—characterized by multisensory disturbances in varying combinations and intensities (e.g., coexisting noise, motion, and visual stimuli)—differentially impact attentional allocation during mobile reading, robustly confirming the roles of environmental complexity and individual interest as key moderators. Methodologically, it develops a dual-method approach integrating objective eye-tracking metrics with subjective interview insights, capturing not only attentional dynamics but also readers’ experiences to comprehensively reveal the mechanisms of environmental distraction. Practically, it delivers actionable strategies for readers, educators, and designers to optimize attention and performance across diverse mobile reading contexts. Collectively, these insights bridge a critical research gap while offering evidence-based pathways to enhance real-world reading engagement.

## Figures and Tables

**Table 1:** Participant Demographic Characteristics (N = 32).

		Frequency	Percentage
Gender	Male	15	46.88%
	Female	17	53.13%
Age	20	10	31.25%
	21	12	37.50%
	22	7	21.88%
	23	3	9.38%
Grade	Sophomore	9	28.13%
	Junior	13	40.63%
	Senior	7	21.88%
	Graduate I	3	9.38%

**Table 2:** External Disturbance Design.

	External Disturbance
Noise Disturbance	Radio Background Noise
	Keyboard Typing Sound
	Traffic Noise
	Construction Noise
	Staff Conversation
Potential Task-like Disturbance	Phone Answering
	Staff-Participant Conversation

**Table 3:** External Scene Design.

	External Scene	Auditory Disturbance	Visual Disturbance	Shaking
Fixed Location	Dormitory	Weak	Weak	None
	Library	Weak	Weak	None
	Cafeteria	Strong	Strong	None
	Entertainment Venue	Strong	Strong	None
	Classroom	Strong	Strong	None
Commuting Scene	Car	Moderate	Moderate	Moderate Shake
	Bus	Moderate	Moderate	Moderate Shake
	Subway	Moderate	Moderate	None

**Table 4:** Specific Experimental Process.

Time	Type	Environment	Operation
00:00–00:30		Quiet	
00:30–01:30	Noise Disturbance	Radio Background Noise	Play sound materials
01:40–02:40		Keyboard Typing Sound	
02:50–03:50		Traffic Noise	
04:00–05:00		Construction Noise	
05:10–06:10		Staff Conversation	On site operation by staff
06:20–07:20	Potential Task-like Disturbance	Phone Answering	
07:30–08:30		Staff-Participant Conversation	
08:30–14:00		Relax	
14:00–16:00	Fixed Location	Dormitory	Play video materials
16:30–18:30		Library	
19:00–21:00		Cafeteria	
21:30–23:30		Entertainment Venue	
24:00–26:00		Classroom	
26:30–28:30	Commuting Scene	Car	
29:00–31:00		Bus	
31:30–33:30		Subway	
34:00–40:00		Interview	On-site operation by staff

Note: The experimental timeline used mm:ss format throughout, where 01:30 denotes 1 min and 30 s.

**Table 5:** Descriptive Statistics and ANOVA Results for Total Fixation Duration Across External Disturbances.

Disturbance	N	M	SD	F	*p*	η^2^_p_
Radio Background Noise	30	34.543	11.290	21.219	0.000	0.423
Keyboard Typing Sound	30	33.950	11.173			
Traffic Noise	30	31.739	11.390			
Construction Noise	30	31.325	12.197			
Staff Conversation	30	31.650	11.829			
Phone Answering	30	25.260	11.193			
Staff-Participant Conversation	30	26.230	11.797			

**Table 6:** Significant Results of Pairwise Comparisons of Total Fixation Duration Among Different External Disturbances.

Disturbance	1	2	3	4	5	6	7
1		1.000	0.111	**0.024**	0.268	**0.000**	**0.000**
2	1.000		**0.027**	**0.014**	0.179	**0.000**	**0.000**
3	0.111	**0.027**		1.000	1.000	**0.001**	**0.017**
4	**0.024**	**0.014**	1.000		1.000	**0.002**	**0.021**
5	0.268	0.179	1.000	1.000		**0.000**	**0.001**
6	**0.000**	**0.000**	**0.001**	**0.002**	**0.000**		1.000
7	**0.000**	**0.000**	**0.017**	**0.021**	**0.001**	1.000	

Note: 1–7 represent external disturbances: Radio Background Noise, Keyboard Typing Sound, Traffic Noise, Construction Noise, Staff Conversation, Phone Answering, Staff-Participant Conversation. Bold font indicates *p* < 0.05.

**Table 7:** Descriptive Statistics and ANOVA Results for Fixation Count Across External Disturbances.

Disturbance	N	M	SD	F	*p*	η^2^_p_
Radio Background Noise	30	190.970	35.388	23.823	0.000	0.451
Keyboard Typing Sound	30	194.200	36.299			
Traffic Noise	30	190.830	40.028			
Construction Noise	30	187.200	49.963			
Staff Conversation	30	180.300	42.590			
Phone Answering	30	150.430	43.170			
Staff-Participant Conversation	30	151.300	42.357			

**Table 8:** Significant Results of Pairwise Comparisons of Fixation Count Among Different External Disturbances.

Disturbance	1	2	3	4	5	6	7
1		1.000	1.000	1.000	0.574	**0.000**	**0.000**
2	1.000		1.000	1.000	**0.006**	**0.000**	**0.000**
3	1.000	1.000		1.000	0.060	**0.000**	**0.000**
4	1.000	1.000	1.000		1.000	**0.001**	**0.000**
5	0.574	**0.006**	0.060	1.000		**0.004**	**0.000**
6	**0.000**	**0.000**	**0.000**	**0.001**	**0.004**		1.000
7	**0.000**	**0.000**	**0.000**	**0.000**	**0.000**	1.000	

Note: 1–7 represent external disturbances: Radio Background Noise, Keyboard Typing Sound, Traffic Noise, Construction Noise, Staff Conversation, Phone Answering, Staff-Participant Conversation. Bold font indicates *p* < 0.05.

**Table 9:** Descriptive Statistics and ANOVA Results for Total Fixation Duration Across External Scenes.

Scene	N	M	SD	F	*p*	η^2^_p_
Dormitory	28	52.399	23.873	3.678	0.015	0.120
Library	28	47.870	21.450			
Cafeteria	28	45.259	19.607			
Entertainment Venue	28	46.708	20.197			
Classroom	28	45.800	19.545			
Car	28	44.798	20.122			
Bus	28	43.104	20.859			

**Table 10:** Significant Results of Pairwise Comparisons of Total Fixation Duration Among Different External Scenes.

Scene	1	2	3	4	5	6	7
1		0.125	**0.034**	0.076	0.071	**0.028**	0.227
2	0.125		1.000	1.000	1.000	1.000	1.000
3	**0.034**	1.000		1.000	1.000	1.000	1.000
4	0.076	1.000	1.000		1.000	1.000	1.000
5	0.071	1.000	1.000	1.000		1.000	1.000
6	**0.028**	1.000	1.000	1.000	1.000		1.000
7	0.227	1.000	1.000	1.000	1.000	1.000	

Note: 1–7 represent external scenes: Dormitory, Cafeteria, Entertainment Venue, Classroom, Library, Car, Bus. Bold font indicates *p* < 0.05.

**Table 11:** Descriptive Statistics and ANOVA Results for Fixation Count Across External Scenes.

Scene	N	M	SD	χ^2^	df	*p*
Dormitory	28	315.180	91.529	13.478	6	0.036
Library	28	294.140	93.098			
Cafeteria	28	280.110	81.436			
Entertainment Venue	28	289.180	82.076			
Classroom	28	285.250	82.743			
Car	28	279.360	88.507			
Bus	28	277.110	100.935			

**Table 12:** Significant Results of Pairwise Comparisons of Fixation Count Among Different External Scenes.

Scene	1	2	3	4	5	6	7
1		1.000	0.057	1.000	0.306	**0.030**	1.000
2	1.000		1.000	1.000	1.000	1.000	1.000
3	0.057	1.000		1.000	1.000	1.000	1.000
4	1.000	1.000	1.000		1.000	1.000	1.000
5	0.306	1.000	1.000	1.000		1.000	1.000
6	**0.030**	1.000	1.000	1.000	1.000		1.000
7	1.000	1.000	1.000	1.000	1.000	1.000	

Note: 1–7 represent external scenes: Dormitory, Cafeteria, Entertainment Venue, Classroom, Library, Car, Bus. Bold font indicates *p* < 0.05.

## Data Availability

The data supporting this study are available from the corresponding author upon reasonable request.

## Note

1 The online access links to the four articles are listed below. Accessed 25 June 2025. https://www.sklib.cn/booklib/directoryPreview?SiteID=122&ID=7142696&fromSubID=&Type=sect1&toDetail=Y; https://weread.qq.com/web/reader/697325f0813ab918bg013bb1kc81322c012c81e728d9d180; https://weread.qq.com/web/reader/17b32d505e393517bbb8f7fkc9f326d018c9f0f895fb5e4; https://weread.qq.com/web/reader/0fa322d0813ab6aacg0159fdk6f4322302126f4922f45dec.

## Abbreviations

The following abbreviations are used in this manuscript:

EEG	Electroencephalogram
CFF	Critical flicker-fusion frequency
FC	Fixation count
TVD	Total visit duration
TTF	Time to first fixation
TFD	Total fixation duration
